# Improving depth-resolution, in-plane contrast, and reducing non-uniformity artifacts for wide-angle DBT

**Published:** 2026-09-22

**Authors:** Emil Y. Sidky, Adrian A. Sanchez, John Paul Phillips, Zheng Zhang, Dan Xia, Ingrid S. Reiser, Xiaochuan Pan

**Affiliations:** a The University of Chicago, Department of Radiology MC-2026, 5841 S. Maryland Ave., Chicago, IL 60615; b Washington University, Mallinckrodt Institute of Radiology, Campus Box 8131-19-01, 510 South Kingshighway Boulevard, Hazelwood, St. Louis, MO 63110

**Keywords:** digital breast tomosynthesis, image reconstruction, sparsity regularization

## Abstract

**Purpose:**

This work aims to develop an image reconstruction algorithm for wide-angle digital breast tomosynthesis (DBT) that has improved depth resolution and in-plane contrast while reducing non-uniformity artifacts.

**Approach:**

The image reconstruction algorithm is an extension of our prior work on sparsity-regularized iterative image reconstruction. The algorithm is performed in two stages as explained in a prior work. The first stage consists of a low-resolution reconstruction that exploits sparsity for quantitative accuracy. In this work, this first stage is augmented with a formulation that includes the estimation of a “background” image, which absorbs low-frequency artifacts that cause image non-uniformity.

**Results:**

The new algorithm is demonstrated on three patient cases, each with different features of clinical interest, and for which the data are acquired on a wide-angle DBT system.

**Conclusion:**

The results on the shown cases indicate that the algorithm design goals have been met, but additional empirical results and task-based assessment are needed to strengthen this conclusion.

## 1 Introduction

The impact of Professor Barrett’s work on our research is profound and multi-faceted and it would be impossible to detail every instance where his influence was felt. Here, in this special issue article, we focus specifically on our recent line of investigation into sparsity-exploiting image reconstruction for digital breast tomosynthesis (DBT). In this project, we have mainly made use of Chapter 7 “Deterministic Description of Imaging Systems” in the *Foundations of Image Science* (FIS),^1^ coauthored by Professor Barrett and Dr. Kyle Myers. Understanding the details of this chapter is crucial to anyone who is interested in developing image reconstruction algorithms, because image reconstruction is essentially an inversion of an imaging system model and thus a clear understanding of the types of image system models is necessary.

The organization of the paper is as follows: Sec. 2 explains how the taxonomy of imaging system models is utilized to formulate our studies on iterative image reconstruction for limited-data systems such as DBT. Sec. 3 then develops an extension to our previous DBT work that addresses depth resolution, in-plane contrast, and non-uniformity artifacts; Sec. 4 presents methods to achieve standard DBT in-plane resolution; Sec. 5 describes the DBT scanner configuration along with the algorithm parameter settings, and Sec. 6 demonstrates the algorithm on three patient cases acquired on a wide-angle DBT system.

## 2 Imaging system models and sparsity-exploiting image reconstruction in digital breast tomosynthesis

Chapter 7 in FIS describes three imaging system models: continuous-to-continuous (CC), continuous-to-discrete (CD), and discrete-to-discrete (DD). For X-ray-based tomography, the divergent-ray and Radon transforms are instances of CC models, where the object model is a continuous function of spatial variables and the measurement space is imagined to be a continuous function of X-ray source angle and detector receptor location. The CD model, however, is a more accurate description for any form of digital imaging because digital detectors consist of discrete arrays of pixels. Finally, the DD model must be contended with because image processing or reconstruction algorithms are implemented on computers, which necessarily discretize images so that they can be stored in memory and manipulated by the various programmed processing units. In addition, fully utilizing Dr. Barrett’s taxonomy of imaging models and his emphasis on task-based image quality assessment is reflected throughout all of our work on imaging.

For analytic image reconstruction, the filtered back-projection (FBP) formulas are derived to be the inverse of the CC divergent-ray or Radon transforms. Because the measured data are discrete, some form of interpolation is implicit when creating the input sinogram to an FBP algorithm. Because interpolation may not perfectly reconstruct the continuous sinogram, the output image values will have error even for ideal, noiseless sinogram measurements. Furthermore, the FBP algorithm can only be executed on a computer for a finite spatial grid. Thus, interpolation is again needed if the display pixel grid does not match the grid of points, which has been reconstructed by FBP. For iterative image reconstruction, the DD model plays a larger role because the scanned object is represented by a finite set of expansion functions, which is most commonly taken to be a grid of voxels. Thus, another source of error is introduced; namely, the divergent-ray transform of a grid of voxels likely does not match the divergent-ray transform of a continuous object function for any set of voxel coefficients. Thus discretization error is an issue with which all iterative algorithms must contend.

Turning to sparsity-exploiting image reconstruction in X-ray tomography, we write the canonical optimization problem for compressive sensing (CS)^2^

(1) $$\underset{f}{\mathrm{arg min}}\|\nabla f{\|}_{1}\;\text{such that}\;Xf=g,$$

where *f* represents the image; *g* is the sinogram; *X* is the X-ray, or divergent-ray, transform; and the objective function is the image total variation (TV). This optimization is designed to recover the image *f* under the assumption that *f* has a sparse gradient and the sinogram data is ideal; namely the data are generated from the true underlying object *f*_0_, i.e. *g* = *Xf*_0_. This CS optimization becomes interesting when the measured data coverage is less than what would be needed for FBP image reconstruction; i.e. the equation *Xf* = *g* does not have a unique solution. As of yet, we have not specified the type of imaging model; this optimization can be expressed in CC, CD, and DD forms. It turns out that the vast majority of the work on CS does in fact assume a DD imaging system model; the conditions for recovery of *f*, discussed in the literature, mainly focus on the number of samples in *g* being some factor larger than the number of non-zeros in the image gradient. Such conditions only make sense with a DD formulation. Accordingly, ∇ becomes a matrix encoding a finite differencing approximation to the continuous gradient; *X* is the discretized X-ray transform; *g* is a set of sinogram samples; and *f* is a vector of image basis coefficients, most commonly voxel values.

For X-ray tomography, the CS optimization problem was originally formulated in CT to address view-angle undersampling, limited angular-range scanning, and cone-beam artifacts from a circular scan,^3, 4^ but as explained in Pan *et al.*^5^ CS can address a number of under-sampling issues such as projection truncation, bad detector pixels, and exterior/interior tomography. An important point to understand about all of these sampling challenges is that their degree of difficulty can be tuned for DD imaging system models. Because much CS work focuses on data sampling, mentioning the image basis expansion in passing or perhaps not at all, it leaves the impression that the sampling challenge is being addressed for CC or CD imaging system models and that a continuous image function – or a close approximation thereof – is being recovered. The reality is, however, that DD imaging system models are being employed, and only a finite set of image basis coefficients are being recovered. With this being the case any sampling challenge can be overcome simply by reducing the size of the basis expansion set. The price to be paid is that discretization error increases when performing image reconstruction on real data or simulation data generated with a continuous image model.

These issues surrounding DD modeling are central to our work on the limited angular-range image reconstruction problem that arises in DBT. Our first steps in accurately solving a DD model for DBT were reported in Zhang *et al.*,^6^ where the optimization problem of interest was a variant of TV-based image reconstruction that used directional TV (DTV) constraints. Following up on this work, a thorough investigation on wide-angle DBT image reconstruction with DTV penalties was presented in Sidky *et al.*^7^ The wide-angle system studied was that of the Siemens Mammomat scanner that has 85 micron detector pixel resolution and scans the breast with 25 exposures covering a 50 degree scan arc. The first study in that paper directly addressed the issue of “tuning” the DD model in order to get accurate image reconstruction for wide-angle DBT. Based on those studies, it was determined that relatively accurate and stable image reconstruction could be performed in a volume composed of cubic voxels with a width four times the width of the detector pixels. The remainder of that work investigated the impact of discretization error when the data are generated from a CD breast phantom model, and additionally the DTV algorithm was applied to DBT image reconstruction for a physical phantom obtained on a Mammomat scanner.

Central to all of these studies is the need for accurate solvers for non-smooth convex optimization problems. Many of the DD imaging system models of interest do not have analytic solutions available and they involve convex optimization problems with non-smooth terms such as the DTV norm or hard constraints such as non-negativity. The only way to provide evidence that the DD imaging system model of interest can be solved is to gather empirical results with computer-simulated phantoms, where the ground truth is known exactly. It must then be demonstrated that the phantom can be recovered perfectly from ideal noiseless data generated with this phantom. For this reason, we have been consistently adapting and refining the primal-dual hybrid gradient (PDHG) algorithm^8^ for X-ray tomographic image reconstruction,^9^ where the latest development is focused on efficient implementation for multi-term optimization problems, which are specifically needed for DD imaging system models relevant to DBT.^10^

The imaging system model design in this latest work is heavily influenced by the ideas discussed in FIS. The overall design is to split the reconstruction problem into two stages: a low-resolution sparsity-exploiting reconstruction using a least-squares (LSQ) data fidelity with DTV regularization (lowres-LSQ-DTV), and high resolution Tikhonov-regularized least-squares (highres-LSQ-Tik). The design goal of lowres-LSQ-DTV is to minimize image root-mean-square-error (RMSE) at low resolution. The low-resolution aspect is needed because ”inverse crime” inversion of the DD imaging model tells us that accurate image reconstruction becomes possible for cubic voxels four times larger than the detector pixels for wide-angle DBT as demonstrated in Refs. 7, 10. To avoid artifacts at this low-resolution both the object model and input data make use of gaussian smoothing that helps to avoid a jagged image representation and to ensure that the data fed into lowres-LSQ-DTV do not contain frequency information that is not representable with the low-resolution image grid; the need for this data filtering becomes clear from the various forms of discretization error explained in Chapter 7 of FIS. The use of large voxels and the fact that lowres-LSQ-DTV is optimized on image RMSE means that small, yet important, features in the image could be lost due to over-regularization – as demonstrated by a signal detection study using the channelized Hotelling observer machinery that was so well-explained in FIS.^11^ To bring in the necessary fine details into the final image, the lowres-LSQ-DTV output image is upsampled to *f*_DTV_ and this image initializes the highres-LSQ-Tik algorithm, where the Tikhonov penalty terms also ensures proximity to *f*_DTV_. Because lowres-LSQ-DTV provides a low RMSE initial image, the parameters of highres-LSQ-Tik can then be objectively determined based solely on clinical tasks of interest to DBT such as the detection of spiculations or microcalcifications using the machinery developed in FIS.

## 3 Coupled lowres-LSQ-DTV image reconstruction for estimating background artifacts

In this section, we present another advance of our DBT image reconstruction framework that deals with low-frequency background artifacts. Such artifacts enter into the raw reconstruction for a variety of reasons: imperfect knowledge of the X-ray illumination in each projection, X-ray scatter, and blurred high-density regions above or below the viewing plane. These low-frequency artifacts complicate display of the in-plane slices because they limit the ability to set the gray scale window for maximizing contrast of features of interest. In our previous work, Ref. 7 reconstructed in-plane slices were post-processed to remove low-frequency artifacts by fitting a low-order 2D polynomial to each slice and dividing the raw slice image by this fit. While effective for image presentation, it is not a 3D solution and it loses quantitative information.

Here, we augment lowres-LSQ-DTV, developed in Ref. 10, to coupled lowres-LSQ-DTV where three images are reconstructed simultaneously. The raw reconstructed image is denoted by *f*_1_; the “background” image, which will primarly contain the low-frequency artifacts, is denoted by *f*_2_; and the difference image is *f*_3_ = *f*_1_ – *f*_2_. The optimization problem for coupled lowres-LSQ-DTV is

(2) $$\underset{f_{1},f_{2},f_{3}}{\text{arg min}}\{\alpha_{x}{\left\|\partial_{x}f_{1}\right\|}_{1}+\alpha_{y}{\left\|\partial_{y}f_{1}\right\|}_{1}+\alpha_{a}{\left\|{\partial_{a}f}_{1}\right\|}_{1}+\alpha_{b}{\left\|{\partial_{b}f}_{1}\right\|}_{1}+\alpha_{1}{\left\|f_{1}\right\|}_{1}\;+\alpha_{x}{\left\|\partial_{x}f_{2}\right\|}_{1}+\alpha_{y}{\left\|\partial_{y}f_{2}\right\|}_{1}+\alpha_{a}{\left\|\partial_{a}f_{2}\right\|}_{1}+\alpha_{b}{\left\|\partial_{b}f_{2}\right\|}_{1}+\alpha_{1}{\left\|f_{2}\right\|}_{1}\;+\alpha_{3}{\left\|f_{3}\right\|}_{1}\;\text{such that}\;f_{1}-f_{2}=f_{3},\;{\left\|R[c]\left(XG\left[d_{1}\right]f_{1}-g\right)\right\|}_{2}\leq \epsilon_{1}\cdot \sqrt{N_{\text{data}}},\;\text{and}\;{\left\|R[c]\left(XG\left[d_{2}\right]f_{2}-G_{\text{det}}\left[d_{d}\right]g\right)\right\|}_{2}\leq \epsilon_{2}\cdot \sqrt{N_{\text{data}}}\},$$

where *g* represents the DBT projection data and *N*_data_ is the size of this dataset; *X* is the DBT X-ray transform that computes a projection data estimate based on an image estimate *f*; the operator *G[d]* performs 3D gaussian blurring with width *d* = (*d_x_, d_y_, d_z_*) and likewise *G*_det_*[d]* performs 2D gaussian blurring on each projections with width *d* = (*d_u_, d_v_*); the operator *R[c]* performs projection filtering with the square root of the ramp filter with frequency cutoff parameter *c*; the parameters $\epsilon_{1}$ and $\epsilon_{2}$ are RMSE constraint parameters on the projection estimate for images *f*_1_ and *f*_2_; the objective function consists of a number of sparsity-inducing $\ell_{1}$-norm terms; the partial derivative symbol represents the finite differencing approximation to the derivative in the direction indicated in the subscript, where *a* and *b* represent oblique directions corresponding to vectors perpendicular to first and last X-ray source position in the *xz*-plane, see Sec. 9; the parameters $\alpha_{x}$, $\alpha_{y}$, $\alpha_{a}$, $\alpha_{b}$, and $\alpha_{1}$ specify the relative strength of each sparsity-inducing term by normalizing the sum of these parameters to 1; note that the same parameters are used for *f*_1_ and *f*_2_; and finally, the parameter $\alpha_{3}$ sets the strength on the $\ell_{1}$ penalty for the difference image *f*_3_. The PDHG algorithm for this optimization is derived by mapping it onto the generic PDHG form; Sec 9 shows how this mapping is done and it specifies the step-size parameterization.

A large part of the image model design leading to Eq. (2) is explained in our previous paper Ref. 10. The new aspect is setting up the model for simultaneous optimization of the raw image *f*_1_ and the “background” image *f*_2_ and coupling these two images through the $\ell_{1}$-norm penalty on *f*_3_. Use of two data fidelity terms with low- and high-frequency bands has the potential to improve algorithm convergence^12^ in addition to the estimation of *f*_1_ and *f*_2_ images. The use of the $\ell_{1}$-norm penalty on *f*_3_ is to encourage small differences between *f*_1_ and *f*_2_ to continue to decrease, and more importantly, the $\ell_{1}$-norm does not strongly penalize large differences. We are expecting that *f*_1_ will deviate from *f*_2_ for in-focus features of the breast. For the data fidelity term involving *f*_2_, the intention for introducing the gaussian blur *G*[*d*_2_] is to smooth out in-plane structures; accordingly the *x* and *y* components of the width vector *d*_2_ should be large, on the order of centimeters, while the depth or *z* component of *d*_2_ should be on the order of the slice thickness. The corresponding blur for projection data, *G*_det_[*d_d_*], should be slightly larger than the in-plane blurring components of *d*_2_ in order to suppress frequency components that would pick up real structures in the breast and so that filtered data can be consistent with the designed imaging model for *f*_2_. For the data fidelity term involving *f*_1_, the purpose of the gaussian blur *G*[*d*_1_] is to smooth the blockiness introduce by the use of large voxels before X-ray projection.

## 4 Achieving standard in-plane DBT resolution

The coupled lowres-LSQ-DTV is designed to greatly improve quantitative accuracy at low-resolution, which has the side benefit of improving depth-resolution. This by itself may have clinical use for dense tissue mapping and refined dose models, but for standard DBT image viewing it is necessary to achieve in-plane resolution close to that of the detector. For improving in-plane resolution, two methods are investigated.

First, we follow the strategy of Ref. 10 and use *f*_1_ to initialize a high-resolution Tikhonov-regularized least-squares (DTVinit-LSQ-Tik) image reconstruction

(3) $$\underset{h}{\mathrm{arg min}}\left\{\frac{1}{2}\|R[c](XG[d]h-g){\|}_{2}^{2}+\alpha_{\mathrm{Tik}\frac{1}{2}}{\left\|G[d]h-h_{0}\right\|}_{2}^{2}\right\},$$

where the high-resolution image and upsampled initial image are represented by *h* and *h*_0_, respectively. Ref. 10 explains how to form the initial image *h*_0_ from the low-resolution image *f*_1_; and the gaussian blurring width for DTVinit-LSQ-Tik is chosen to be the same as the voxel dimensions. The algorithm consists of steepest descent steps on the LSQ-Tik objective function. The parameter *α*_Tik_ controls the image regularization. In order to form the final image for display, the background image *f*_2_ is upsampled to the high-resolution background image *h*_2_. The support image, *h*_sup_, indicating voxels inside and outside of the breast with 1 and 0, respectively, is found by thresholding *h*_2_ at a gray level of 0.1 cm^−1^. A mean background gray value, *b*, is found by averaging *h*_2_ in the breast support, i.e. where *h*_sup_ = 1. The image for display is then formed by

(4) $$h_{\text{disp-LSQ}}=G_{z}\left[d_{z}\right]\left(\left(h-h_{2}\right)+b\cdot h_{\text{sup}}\right),$$

where additional depth blurring with a one-dimensional gaussian kernel is introduced for additional regularization.

Second, the coupled lowres-LSQ-DTV result is reprojected and subtracted from the original DBT projection data. This difference is reconstructed with FBP to form an image that contains only the high-resolution detail, which can subsequently be combined with the coupled lowres-LSQ-DTV result. The processing for the high-resolution DTV-FBP algorithm starts with forming the high-resolution difference image

(5) $$h_{\text{diff}}=\mathrm{FBP}\left(g-XG\left[d_{1}\right]h_{1}\right),$$

where the high-resolution image *h*_1_ is obtained by upsampling the raw coupled lowres-LSQ-DTV image, *f*_1_; the Gaussian blurring *G*[*d*_1_] uses a width *d*_1_ matched to the value employed for Eq. (2). In our implementation of FBP, a Hanning filter is used with cutoff set to the Nyquist frequency. For this method, the display image is formed slightly differently than what is described in Eq. (4).

(6) $$h_{\text{disp-FBP}}=G_{z}\left[d_{z}\right]\left(\left(h_{1}+\eta \cdot h_{\text{diff}}-h_{2}\right)+b\cdot h_{\text{sup}}\right),$$

where the combination parameter *η* needs to be tuned. The useful scale for *η* can be determined by reprojecting *h*_diff_ and normalizing to the magnitude of the projection data difference, $g-XG\left[d_{1}\right]h_{1}$.

## 5 Scanner setup and algorithm parameter settings

The algorithm parameter settings depend on the configuration of the DBT scanner, which can vary dramatically from vendor to vendor. For this work, patient data from the Siemens Mammomat B.brilliant scanner is reconstructed. The scan configuration involves 25 X-ray projections covering an arc of 50 degrees, which is currently the widest angle DBT on the market. The X-ray detector is 3584×2816 pixels with each pixel being 85 microns squared. The long-axis of the detector *u* is parallel to the direction of the scan and the *x*-axis of the image volume, while the short-axis *v* is parallel to the *y*-axis of the image volume. To process the transmission data to projection data, the air scan fluence, *I*_0_(*u, v*), is estimated from a strip on the detector well beyond the breast projection. The *u* dependent fluence, ${\overset{‾}{I}}_{0}(u)$, is estimated by averaging *I*_0_(*u, v*) over *v* only within this strip. The estimated projection data, *g*(*u, v*), are obtained by standard negative logarithm processing; i.e. $g(u,v)=-ln\left(I(u,v)/{\overset{‾}{I}}_{0}(u)\right)$.

The coupled lowres-LSQ-DTV image reconstruction is carried out on a low-resolution voxel grid with voxels of size 340x340x680 microns cubed, and the projection data are then down-sampled to a pixel grid with pixels of size 340x340 microns squared. The high-resolution LSQ-Tik and DTV-FBP algorithms use voxels of size 85x85x340 microns cubed, and the native 85x85 microns squared detector resolution for the projections is maintained.

The regularization parameter settings with which coupled lowres-LSQ-DTV is solved are

$$\alpha_{y}=\alpha_{a}=\alpha_{b}=\alpha_{1}=1/9;\;\alpha_{x}=5/9,$$

which are the same values determined by DBT simulations on the same wide-angle DBT system, performed in Ref. 10, and the coupling parameter for the difference image *f*_3_ is set to

$$\alpha_{3}=0.2,$$

which provides a good visual fit to the image background for the three patient cases shown in Sec. 6. The blur parameters *d*_1_, *d*_2_, and *d_d_* from the data discrepancy constraints of Eq. (2) are set to

$$d_{1}=(340, 340, 680)\;\mu m,\;d_{2}=(3400,3400,680)\;\mu m,\;\text{and}\;d_{d}=(3740,3740)\;\mu m,$$

for all three patient cases. That the same parameters can be applied to each of these cases is an indication that the imaging is not so sensitive to these parameter settings.

The data RMSE constraints, on the other hand, are more sensitive and these should be tuned on a case-by-case basis. Here, these parameters are set by visual inspection, but for clinical application an automated procedure must be devised to establish good settings. The settings for the three cases are listed in Table 1. As stated, these data RMSE constraints are the most critical parameters of coupled lowres-LSQ-DTV as they are the primary controls on the image regularization. The looser, i.e. larger, these values are, the more regularized *f*_1_ and *f*_2_ become. The minimum values for $\epsilon_{1}$ and $\epsilon_{2}$ can be found by setting these parameters to zero, then noting what data discrepancy is actually achieved when coupled lowres-LSQ-DTV is run to convergence. Backing off from these minimum values regularizes their respective images *f*_1_ and *f*_2_. Visually, the ϵ values are increased so that speckle noise is removed from *f*_1_ and blobby artifacts on the scale of *d*_2_ are removed from *f*_2_. The depth blur parameters involved in Eqs. (4) and (6) depend on the features of interest.

Finally, the PDHG algorithm parameters for coupled lowres-LSQ-DTV are set to the same values for each of the three cases, as they also do not need to be finely tuned,

γ=5,ρ=1.75,andβ=100;

these parameters are explained in Sec. 9.

## 6 Results on wide-angle DBT image reconstruction for three patient cases

We demonstrate the application of coupled lowres-LSQ-DTV on three patient cases acquired on a Siemens Mammomat B.brilliant scanner. Approval of the Institutional Review Board (IRB) of Washington University (Protocol ID: 202410112) was obtained for this retrospective, Health Insurance Portability and Accountability Act-compliant study conducted from data found in institutional patient databases. Written informed consent was waived by the IRB. Three cases are selected that display different features of clinical interest: a solid mass, an architectural distortion, and a calcification cluster.

Results for coupled lowres-LSQ-DTV are shown in Fig. 1, where an in-plane slice image is shown that contains a suspicious mass near the chest wall. The line profile and difference image show how *f*_2_ is able to model the image background without following clinically relevant features such as the mass, vessels, or fibroglandular tissue; additionally, *f*_2_ is able to account for background low-frequency artifacts. Coupled lowres-LSQ-DTV is also applied to a patient case with a distortion and the results are shown in Fig. 2. The feature of interest, the architectural distortion, is better visualized in the high-resolution region-of-interest (ROI) images shown in Fig. 6, but Fig. 2 shows how the background *f*_2_ image picks up on the uniform dense region just inside the nipple, allowing for the difference image *f*_3_ to highlight fine structures in this region. Finally, the same algorithm is applied to a case with a significant microcalcification cluster, and results are displayed in Fig. 3. Of particular interest is that coupled lowres-LSQ-DTV is able to account for the truncation artifacts at the top of the image, and consequently these artifacts are greatly reduced in the *f*_3_ difference image. The ROI focusing on the calcifications is shown in Fig. 8.

In order to obtain standard DBT resolution, we employ DTVinit-LSQ-Tik as described in Sec. 4. The results of DTVinit-LSQ-Tik are compared with zeroinit-LSQ-Tik, which is found by the same algorithm except that the initial image *h*_0_ has all voxel values set to zero. No background artifact removal for zeroinit-LSQ-Tik is performed. Example in-plane slice images are shown in Fig. 4. Because the background low-frequency artifacts are removed from DTVinit-LSQ-Tik, it is possible to display the image in a tighter grayscale window than the image for zeroinit-LSQ-Tik. Interestingly, the improved depth-resolution afforded by the coupled lowres-LSQ-DTV initial image resolves the tangle of glandular tissue in the outer breast (image top) and improved in-plane contrast highlights the mass at the chest wall.

A comparison between DTVinit-LSQ-Tik and zeroinit-LSQ-Tik for the distortion case is shown in Fig. 5. Once again the the former algorithm is seen to remove much of the display slice clutter due to structure outside of the viewing plane. The removal of low-frequency gray scale variation allows a tight display window thereby improving contrast of important features of clinical interest such as the architectural distortion. A blow-up of this feature is shown in Fig. 6 along with a different high-resolution image generated by high-resolution DTV-FBP. Recall that DTVinit-LSQ-Tik and DTV-FBP images are generated by Eq. (4) and Eq. (6), respectively. In this figure, the initial coupled lowres-LSQ-DTV ROI is shown so that the increase in resolution by either high-resolution DTVinit-LSQ-Tik or DTV-FBP can be appreciated. Both of these results are compared against their counterpart algorithms, zeroinit-LSQ-Tik and FBP respectively, where the advantage in distortion contrast and reduced clutter can be clearly seen. The two alternative algorithms are presented because there is a clear difference in noise texture and image sharpness at this level of magnification.

High-resolution images for the case with micro-calcifications are shown in Fig. 7. As before there is less clutter in the DTVinit-LSQ-Tik image; the new aspect highlighted by this case is that the truncation artifacts seen at the top left in the zeroinit-LSQ-Tik image is effectively suppressed in the DTVinit-LSQ-Tik image. Focusing on the microcalcifications, ROI images for this case are shown in Fig. 8. The difference between the low-resolution ROI and the high-resolution images for this ROI is greater than was the case for the distortion images, due the the fact that calcifications are smaller features. As before, there is a difference in noise texture and sharpness between the high-resolution DTVinit-LSQ-Tik and DTV-FBP images. Use of coupled lowres-LSQ-DTV, however, does not have a large impact for microcalcification imaging as there is not a large difference between the middle and right columns in terms of micro-calcification visualization.

## 7 Discussion

We have demonstrated, with three patient cases, the use of coupled lowres-LSQ-DTV in increasing depth-resolution and slice uniformity for improving contrast and visualization of solid masses and spiculations indicating an architectural distortion. Visualization of micro-calcifications is also shown, but little change in their conspicuity is noted, because existing image reconstruction techniques can already resolve such features well. The proposed two-step reconstruction: using accurately solved coupled lowres-LSQ-DTV followed by either high-resolution FBP or LSQ-Tik, with truncated iteration, is highly efficient. In the shown examples, the low-resolution problem is a factor of 512 smaller than the high-resolution problem, because the volume is represented on a grid with *x* and *y* dimensions reduced by a factor of four each and the *z* dimension by a factor of two. Additionally, the projection dimensions *u* and *v* are reduced by a factor of four each. The computationally demanding steps are X-ray projection and back-projection. The low-resolution coupled lowres-LSQ-DTV algorithm executes a total of 4,000 of these projection operations for the 1,000 iterations used to achieve accurate solution; each iteration has two projection and back-projection operations due to the simultaneous processing of *f*_1_ and *f*_2_ images. The high-resolution LSQ-Tik algorithm is run for 10 iterations, involving 20 projection operations. But because of the factor of 512 gain in efficiency from the reduced grid size, the coupled lowres-LSQ-DTV phase takes half of the computational effort of high-resolution LSQ-Tik. Of course, high-resolution DTV-FBP requires much less computational than effort than LSQ-Tik because it involves only a single back-projection operation.

While the proposed algorithms are seen to be effective in improving depth-resolution and in-plane contrast, there are a few directions of investigation that would demonstrate, or further improve, clinical utility. Of immediate concern is the bright skin-line; we have shown the direct output of the developed image reconstruction algorithms without any post-processing. Skin-line correction is, however, an important post-processing step that is needed for clinical use. Another point is to devise non-linear image reconstruction in the second high-resolution stage specifically for denoising and improving micro-calcification conspicuity,^13^ noting that this work by Zheng *et al.* was performed on a wide-angle system with a 60 degree scan arc. Finally, a larger study of patient cases is needed where the imaging properties can be studied as a function of breast density category.

## 8 Conclusion

We have taken the opportunity of this special issue honoring Dr. Barrett to report on the latest developments on our work in digital breast tomosynthesis image reconstruction. The goal of the algorithm design is to improve depth resolution, in-plane contrast, and to remove non-uniformity artifacts. While the results from the shown clinical cases show promise, it is clear that more studies are needed to demonstrate improved utility. Much of the future effort in this direction will involve carrying out image quality studies promoted by Dr. Barrett; DBT simulations for clinical tasks sensitive to image reconstruction algorithms and parameters, e.g. detection of subtle signals such as microcalcifications or spiculations on the margin of a suspicious mass, will be formulated and employed to assess the algorithms in an objective manner, as we have done in Refs. 14, 15.

The contributions Dr. Barrett has made permeate modern image science. The impact of his work goes far beyond the usual metrics of academic productivity, i.e. number of citations or successful grant applications. His work has been so foundational that it often goes uncited because it is part of the bedrock of the field. Thus we are grateful for the opportunity to spell out, in this work on DBT, the many ways that his contributions have influenced our work.

## Figures and Tables

**Fig 1:**
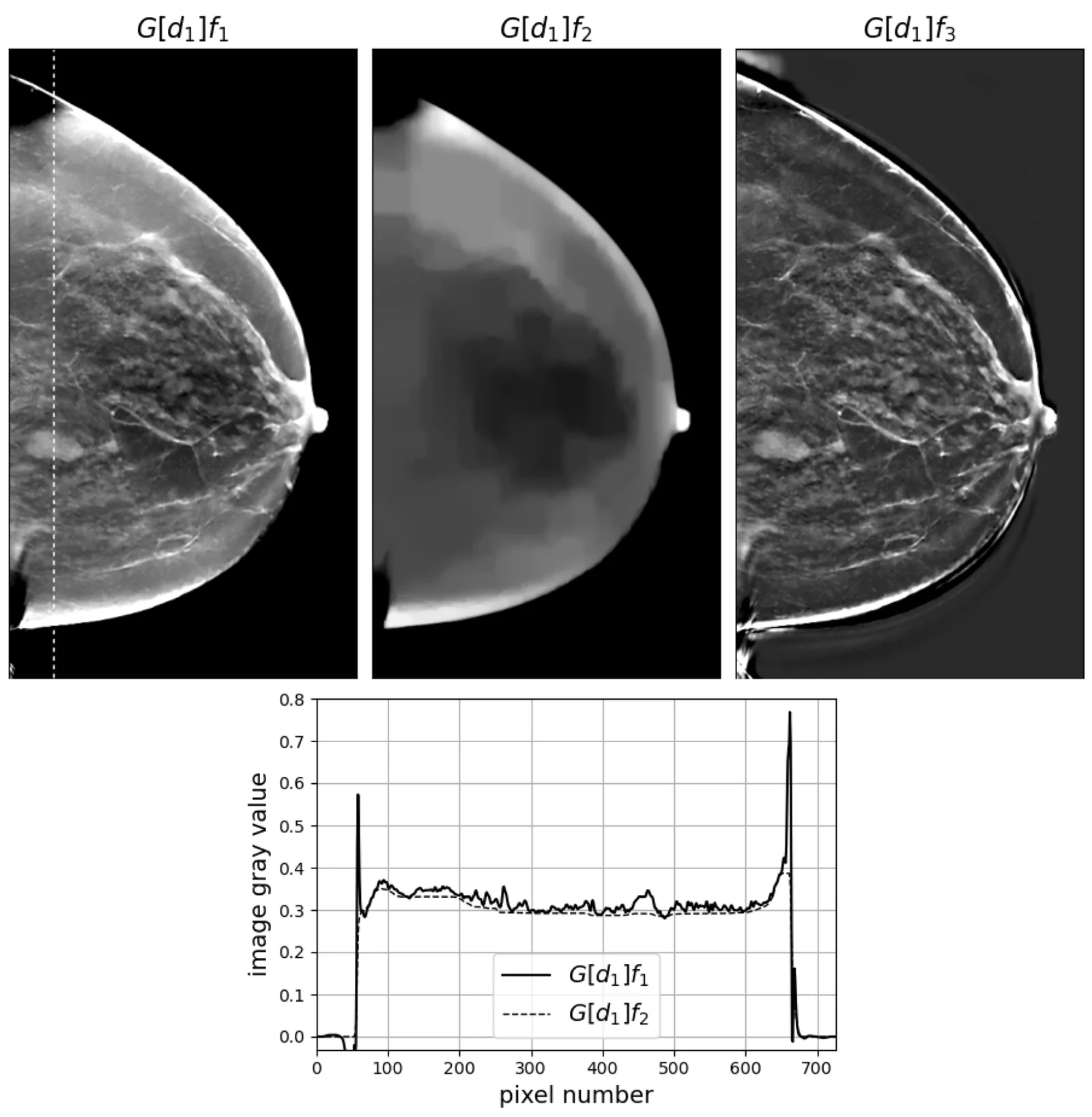
In-plane slice images for the coupled lowres-LSQ-DTV alongside a profile plot demonstrating the effectiveness of *f*_2_ at extracting the image background. The dashed line in the *f*_2_ image indicates the location of the profile plot data.

**Fig 2:**
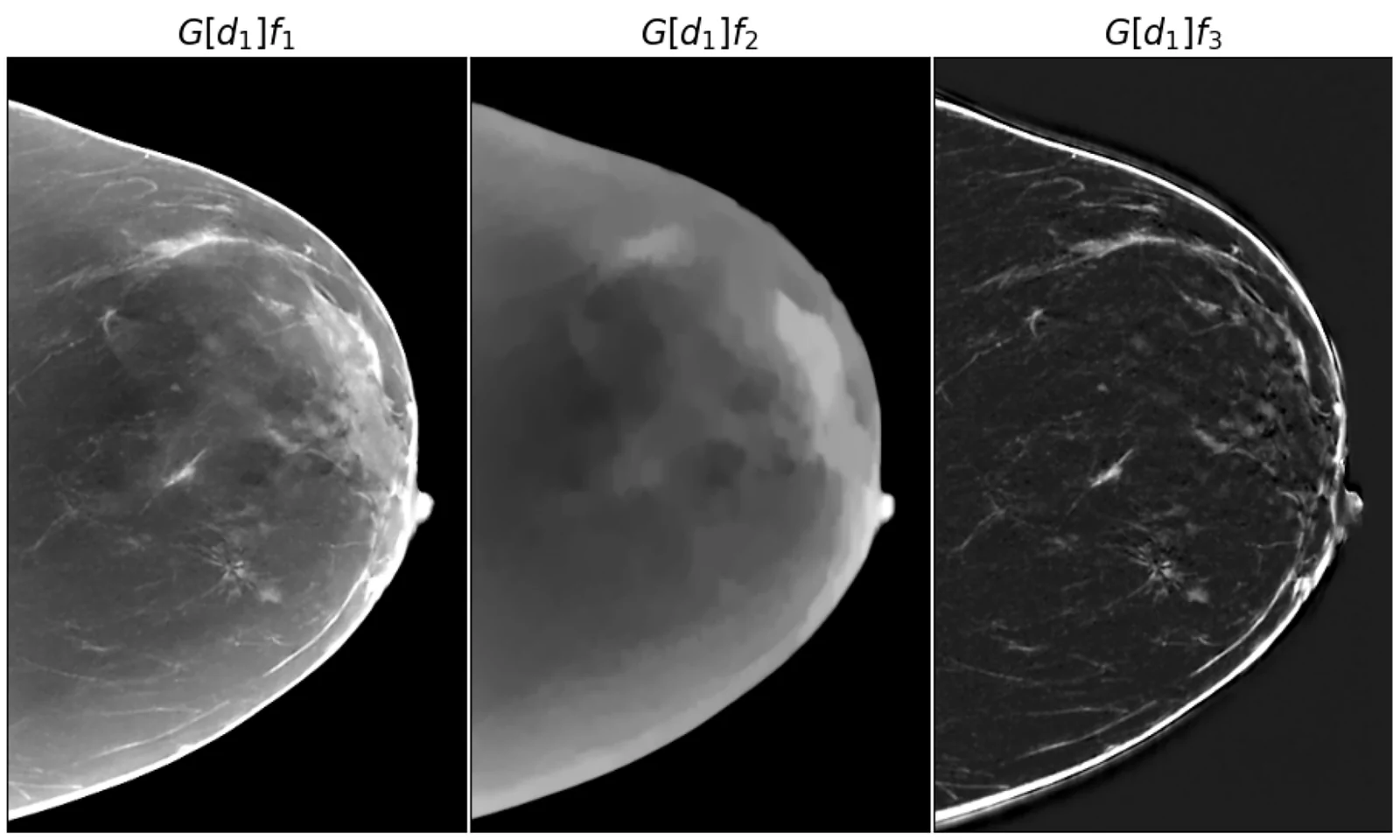
In-plane slice images for the coupled lowres-LSQ-DTV for the patient case containing an architectural distortion.

**Fig 3:**
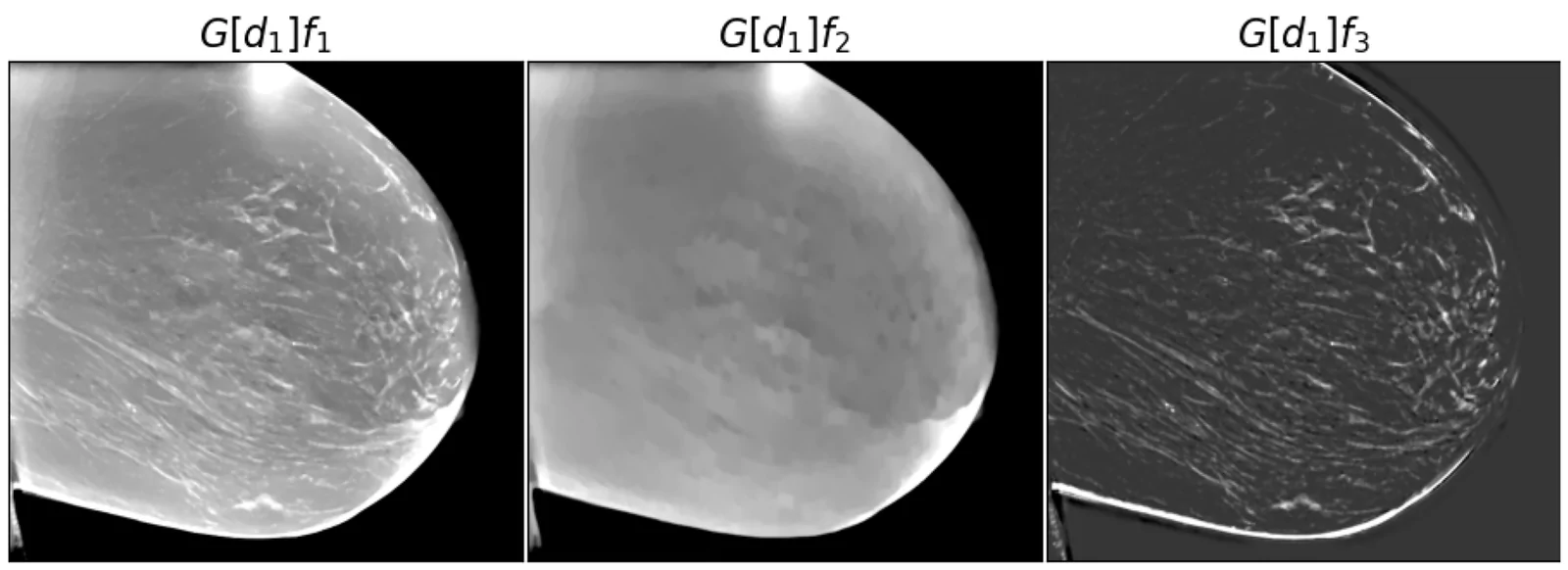
In-plane slice images for the coupled lowres-LSQ-DTV for the patient case containing a calcification cluster.

**Fig 4:**
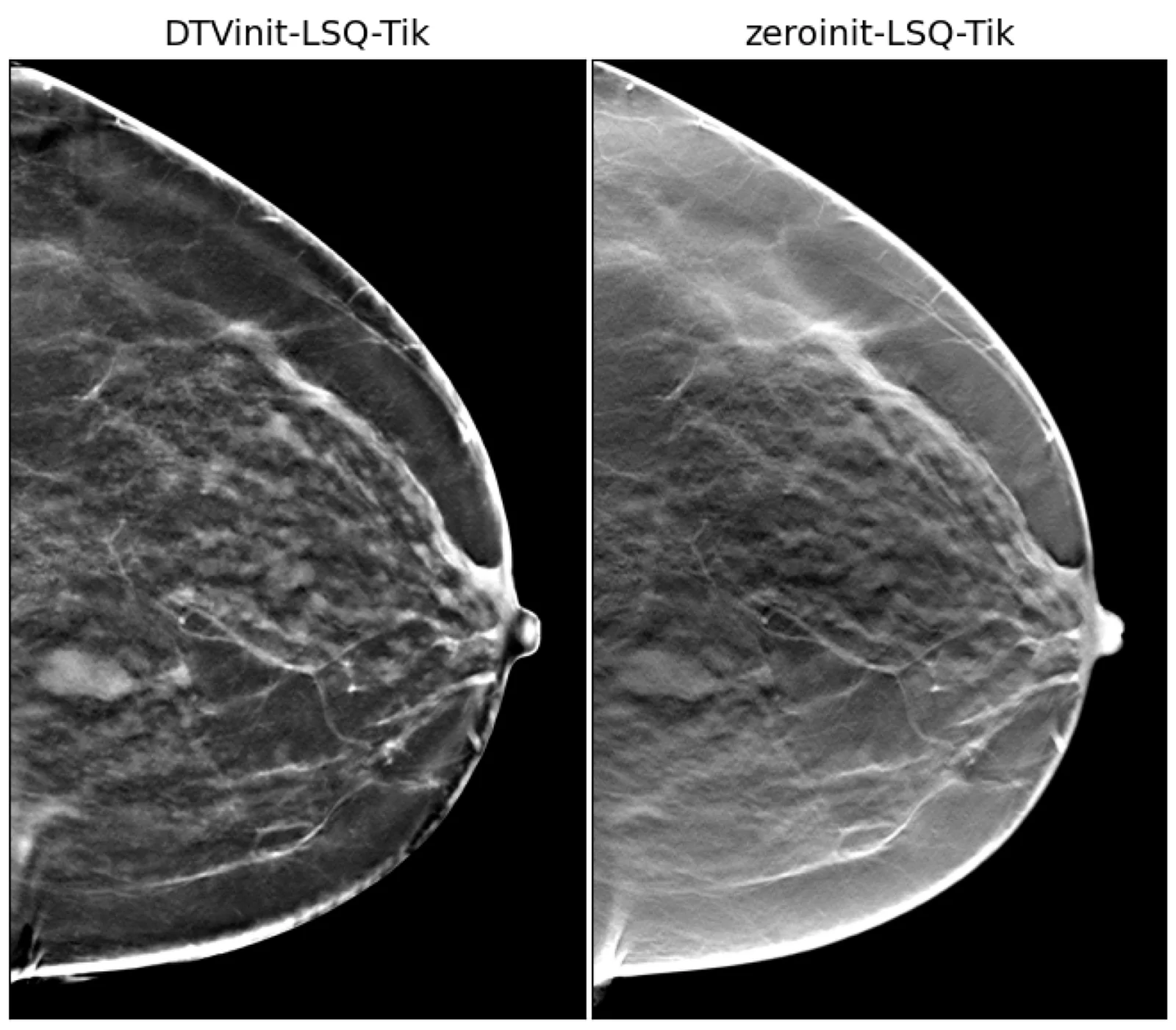
In-plane images of DTVinit-LSQ-Tik (left) and zeroinit-LSQ-Tik (right) for the same slice shown in Fig. 1.

**Fig 5:**
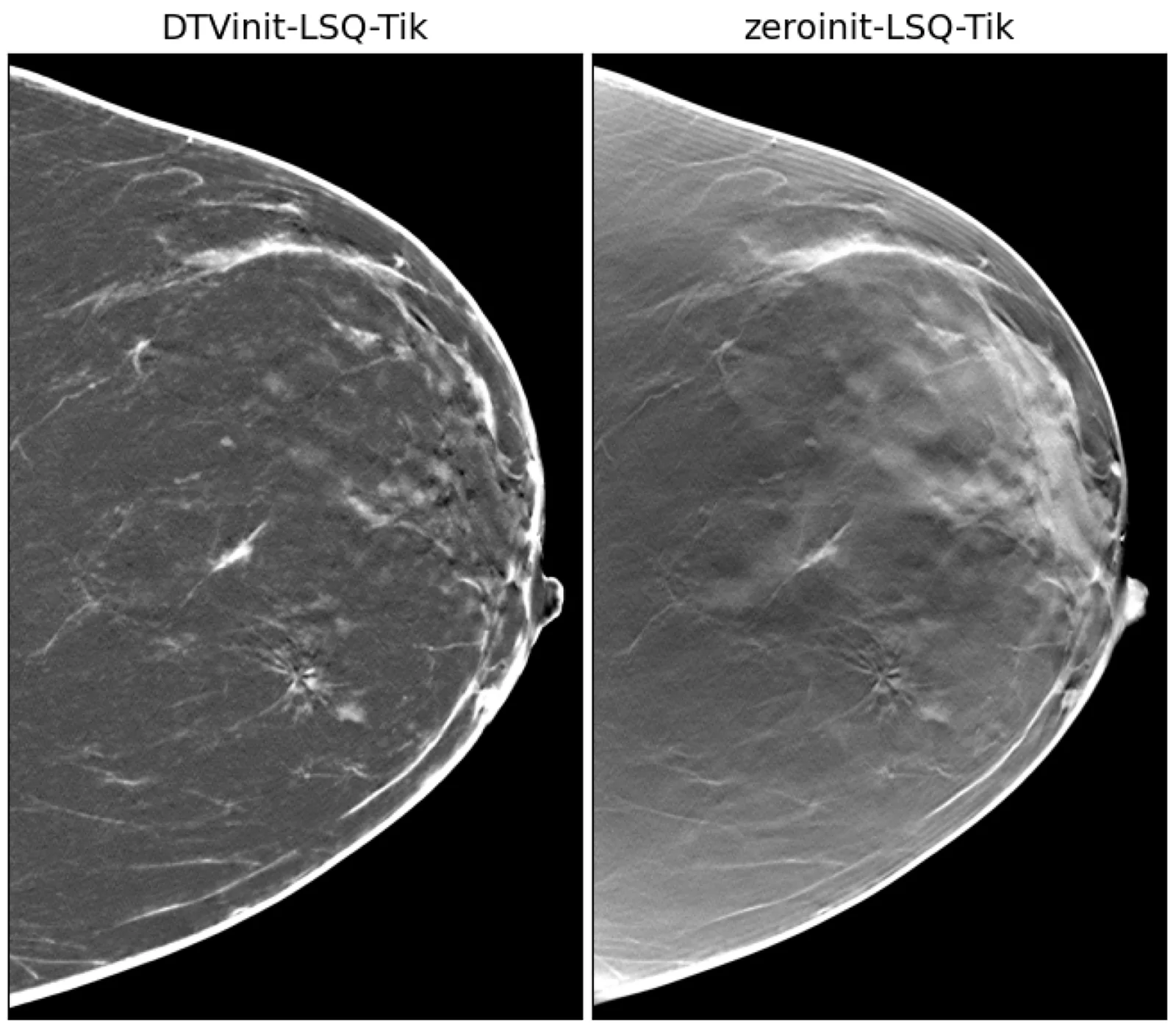
In-plane images of DTVinit-LSQ-Tik (left) and zeroinit-LSQ-Tik (right) for the same slice shown in Fig. 2.

**Fig 6:**
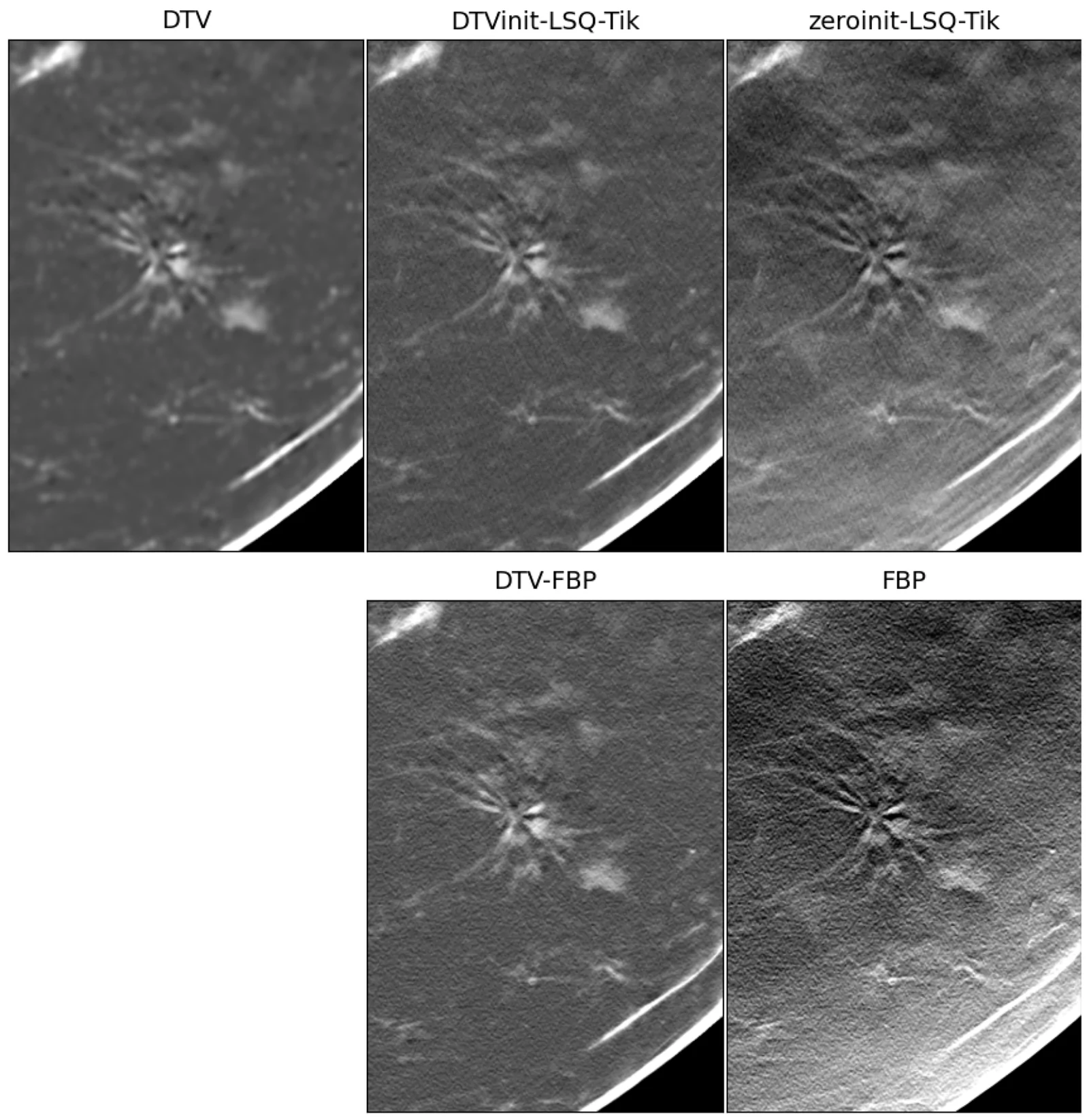
Blow-up ROIs of the architectural distortion from the case displated in Fig. 5. The single image in the left column shows the input coupled lowres-LSQ-DTV result. The middle column shows the results from the proposed high-resolution algorithms. The right column shows the ROIs for the comparison algorithms.

**Fig 7:**
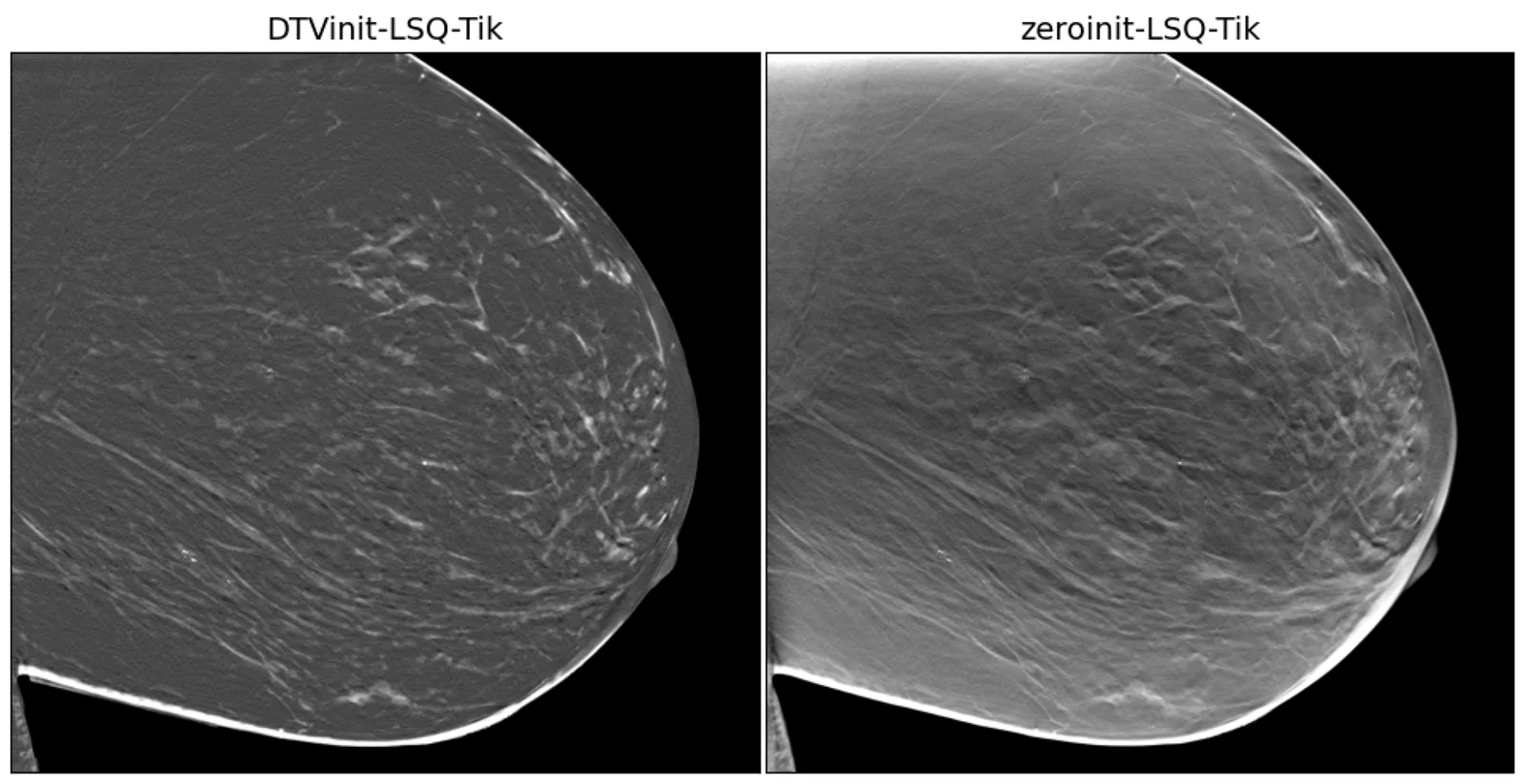
In-plane images of DTVinit-LSQ-Tik (left) and zeroinit-LSQ-Tik (right) for the same slice shown in Fig. 3.

**Fig 8:**
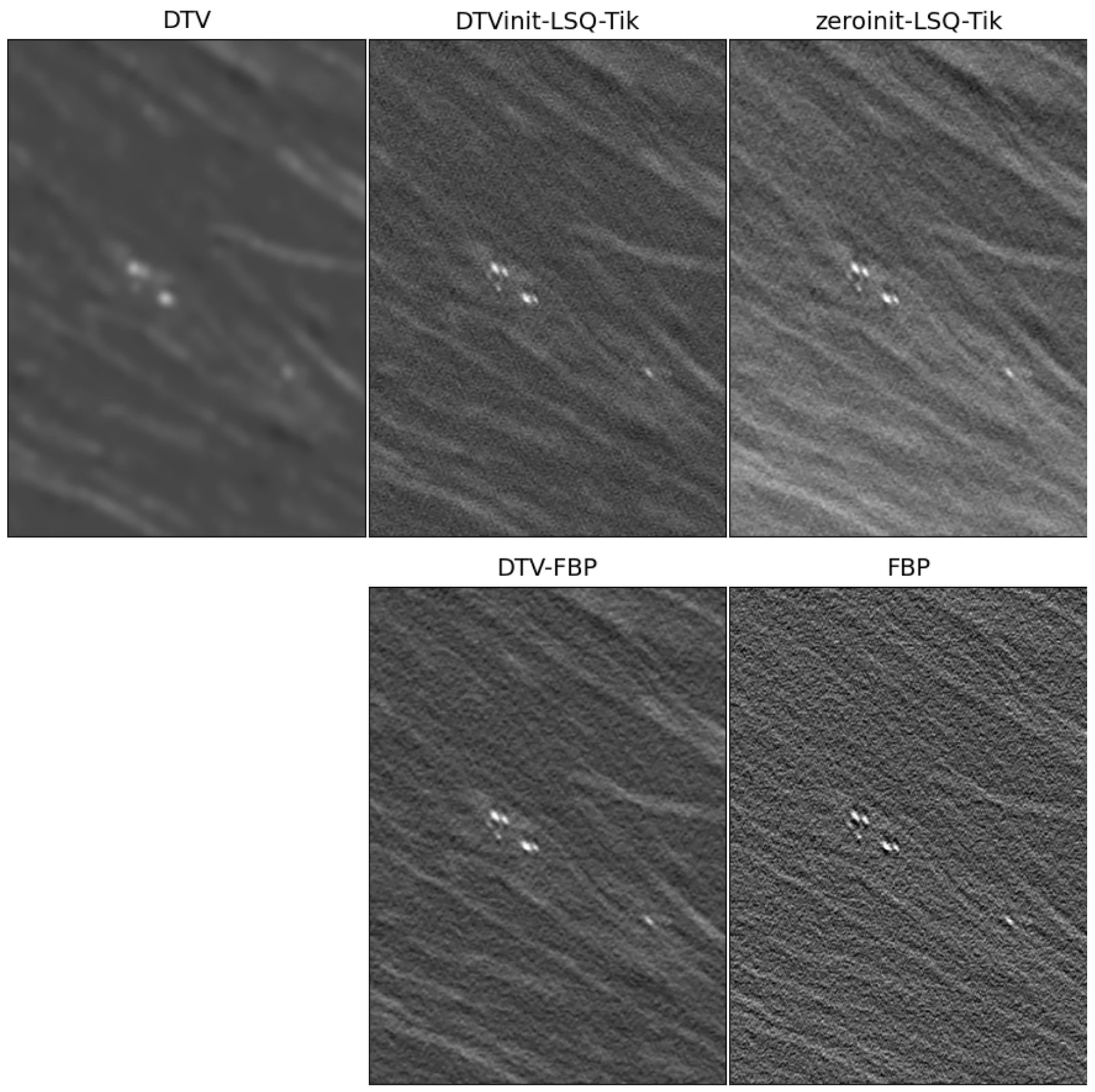
Blow-up ROIs of a calcification cluster from the case displated in Fig. 7. The ROI panel presentation is the same as that of Fig. 6.

**Table 1:** Parameter settings for the coupled lowres-LSQ-DTV optimization and image display depth blurring for each of the patient cases. The cases presented in order contain a solid mass (mass), an architectural distortion (distortion), and a calcification cluster (calcs).

case	*ϵ*_1_	*ϵ*_2_	*d_z_*
mass	0.015	0.015	3400 *μ*m
distortion	0.012	0.01	2040 *μ*m
calcs	0.02	0.0125	1360 *μ*m

## Code, Data, and Materials Availability

There is no code or data in this work for sharing.

## 9 Appendix: PDHG implementation of Eq. (2)

This appendix shows how to fit Eq. (2) into the generic PDHG form

(7)
$$\underset{x}{min}\{F(Kx)+G(x)\},$$

where the *F* and *G* functions are convex and possibly non-smooth; *K* is a linear transforms; and *x* is the unknown vector. With a multiterm objective function such as the one presented in Eq. (2) the function *F* ends up representing a sum of convex functions and *K* is formed by stacking multiple matrix blocks, which are the linear transforms inside of each of the objective terms. As shown in Sec. 2 of Ref. 10, we make the following associations

$$x=\left[\begin{array}{c} f_{1}\\ f_{2}\\ f_{3} \end{array}\right],\;y=\left[\begin{array}{c} y_{1}\\ y_{2}\\ \vdots \\ y_{13} \end{array}\right]=Kx,\;\hat{F}(y)=\overset{13}{\underset{i=1}{\sum }}{\hat{F}}_{i}\left(y_{i}\right)=\overset{13}{\underset{i=1}{\sum }}F_{i}\left(y_{i}{\left\|K_{i}\right\|}_{2}/\nu_{i}\right),$$

$$K=\left[\begin{array}{ccc} \nu_{1}K_{1}/{\left\|K_{1}\right\|}_{2} & \cdot & \cdot \\ \nu_{2}K_{2}/{\left\|K_{2}\right\|}_{2} & \cdot & \cdot \\ \vdots & \vdots & \vdots \\ \nu_{6}K_{6}/{\left\|K_{6}\right\|}_{2} & \cdot & \cdot \\ \cdot & \nu_{7}K_{7}/{\left\|K_{7}\right\|}_{2} & \cdot \\ \cdot & \nu_{8}K_{8}/{\left\|K_{8}\right\|}_{2} & \cdot \\ \vdots & \vdots & \vdots \\ \cdot & \nu_{12}K_{12}/{\left\|K_{12}\right\|}_{2} & \cdot \\ \cdot & \cdot & \nu_{13}K_{13}/{\left\|K_{13}\right\|}_{2} \end{array}\right],$$

where ${\left\|K_{i}\right\|}_{2}$ is the matrix norm, i.e. largest singular value, of the matrix *K_i_* and *v_i_* are matrix scaling parameters that can be optimized for PDHG convergence efficiency. The function and matrix block assignments are as follows

$$F_{1}\left(y_{1}\right)=\delta \left(y_{1}|{\left\|y_{1}-R[c]g\right\|}_{2}\leq \epsilon_{1}\cdot \sqrt{N_{\text{data}}}\right),K_{1}=R[c]XG\left[d_{1}\right]\;F_{7}\left(y_{7}\right)=\delta \left(y_{7}|{\left\|y_{7}-R[c]G_{\text{det}}\left[d_{d}\right]g\right\|}_{2}\leq \epsilon_{2}\cdot \sqrt{N_{\text{data}}}\right),K_{7}=R[c]XG\left[d_{2}\right],\;F_{2}\left(y_{2}\right)=\alpha_{x}{\left\|y_{2}\right\|}_{1},K_{2}=\partial_{x},F_{8}\left(y_{8}\right)=\alpha_{x}{\left\|y_{8}\right\|}_{1},K_{8}=\partial_{x},\;F_{3}\left(y_{3}\right)=\alpha_{y}{\left\|y_{3}\right\|}_{1},K_{3}=\partial_{y},F_{9}\left(y_{9}\right)=\alpha_{y}{\left\|y_{9}\right\|}_{1},K_{9}=\partial_{y},\;F_{4}\left(y_{4}\right)=\alpha_{a}{\left\|y_{4}\right\|}_{1},K_{4}=\partial_{a},F_{10}\left(y_{10}\right)=\alpha_{a}{\left\|y_{10}\right\|}_{1},K_{10}=\partial_{a},\;F_{5}\left(y_{5}\right)=\alpha_{b}{\left\|y_{5}\right\|}_{1},K_{5}=\partial_{b},F_{11}\left(y_{11}\right)=\alpha_{b}{\left\|y_{11}\right\|}_{1},K_{11}=\partial_{b},\;F_{6}\left(y_{6}\right)=\alpha_{1}{\left\|y_{6}\right\|}_{1},K_{6}=I,F_{12}\left(y_{12}\right)=\alpha_{1}{\left\|y_{12}\right\|}_{1},K_{12}=I,\;F_{13}\left(y_{1}3\right)=\alpha_{3}{\left\|y_{13}\right\|}_{1},K_{13}=I,\;G(x)=\delta \left(\left[f_{1},f_{2},f_{3}\right]|f_{1}-f_{2}-f_{3}=0\right),$$

where the indicator function δ(x∣C) is 0(∞) if *x* does (does not) satisfy the equality or inequality *C*; the oblique directional derivatives denoted with *a* and *b* are a combination of partials in the *x* and *z* direction

$$\partial_{\theta }=\cos\theta \partial_{x}+\sin\theta \partial_{z},$$

where *a* and *b* denote the directions *θ* = −25° and 25°, respectively.

**Algorithm 1:** PDHG update steps for solving Eq. (7). The normalized matrices ${\overset{ˆ}{K}}_{i}=K_{i}/{\left\|K_{i}\right\|}_{2}$ are used for conciseness.

1: $x^{\left(k+1\right)}\leftarrow (I+\tau \partial G{)}^{-1}\left(x^{\left(k\right)}-\tau \left(\sum_{i}\nu_{i}{\overset{ˆ}{K}}_{i}^{⊤}\lambda_{i}^{\left(k\right)}\right)\right)$
2: $\tilde{x}=2x^{\left(k+1\right)}-x^{\left(k\right)}$
3: $\lambda_{i}^{\left(k+1\right)}\leftarrow {\left(I+\sigma_{i}\partial {\overset{ˆ}{F}}_{i}^{⋆}\right)}^{-1}\left(\lambda_{i}^{\left(k\right)}+\sigma_{i}\nu_{i}{\overset{ˆ}{K}}_{i}\tilde{x}\right)\;\forall i$

As explained in Ref. 10, the algorithm for solving Eq. (7) involves three update steps, which are repeated in Algorithm 1 for completeness. Lines 1 and 3 of this algorithm involve possibly unfamiliar notation which is fully explained in Ref. 10; the reason why the update steps are given is to specify the complete set of step-size parameters. Line 1 of Algorithm 1 involves the scalar step-size *τ* and the matrix scaling parameters *ν_i_*; Line 3 involves the scalar step-sizes *σ_i_*. These step-size parameters are chosen so that

(8) $$\tau {\left\|\sum_{i}\sigma_{i}\nu_{i}^{2}{\overset{ˆ}{K}}_{i}^{⊤}{\overset{ˆ}{K}}_{i}\right\|}_{2}=1,$$

which is based on the convergence condition for PDHG. Because the index *i* runs through all 13 matrix blocks, there are a total of 27 parameters (*τ*, *σ_i_*, and *ν_i_*) to tune for optimizing the PDHG convergence rate. The condition in Eq. (8) reduces this number to 26 independent parameters, but tuning 26 parameters is still a daunting task.

In Ref. 10, a framework for X-ray tomography is proposed that simplifies the parameter selection. In that work, it is proposed to provide heuristic arguments for selecting step-size weight parameters, ${\overset{ˆ}{w}}_{i}$, and ratio parameters *r_i_*, which are related to *τ*, *σ_i_*, and *ν_i_* by the equations

$$w_{i}=w{\overset{ˆ}{w}}_{i}=\tau \sigma_{i}\nu_{i},\;\text{and}\;r_{i}=\sigma_{i}/\tau ,$$

where ${\overset{ˆ}{w}}_{i}$ are relative weights and the scalar magnitude *w* is determined by Eq. (8)

(9) $$w={\left\|\underset{i}{\sum }{\hat{w}}_{i}{\hat{K}}_{i}^{⊤}{\hat{K}}_{i}\right\|}_{2}^{-1}.$$

In Ref. 10, it is proposed to set all step-size ratios equal to a single scalar *β* and to weight all linear transforms *K_i_* the same except for the matrix blocks involving the X-ray transform, which should have greater weighting *γ*. For Eq. (2) there are two matrix blocks, *K*_1_ and *K*_7_, that involve the X-ray transform. Accordingly, we make the following assignments

$${\overset{ˆ}{w}}_{1}={\overset{ˆ}{w}}_{7}=\gamma ,\;{\overset{ˆ}{w}}_{i}=1,\forall i\neq 1\;\text{or}\;7,\;r_{i}=\beta ,$$

where *γ* is selected in the interval γ∈[1,∞], and *β* is a positive real number. As a result, the search space for the step-size parameters is reduced from 26 dimensions down to 2, which is much more manageable. As is done in Ref. 10, the reduction in step-size parameter search space allows the use of an accelerated predictor-corrector PDHG algorithm that comes with an additional extrapolation parameters *ρ*. The particular instatiation of the PDHG algorithm calls for derivation of proximal operators involving the function terms *F_i_*(*y_i_*) and *G*(*x*) and the details on how to derive those steps are presented in Ref. 10.
